# Genomic Analyses of Metaplastic or Sarcomatoid Carcinomas From Different Organs Revealed Frequent Mutations in KMT2D

**DOI:** 10.3389/fmolb.2021.688692

**Published:** 2021-07-15

**Authors:** Biqiang Zheng, Zhijian Song, Yong Chen, Wangjun Yan

**Affiliations:** ^1^ Department of Musculoskeletal Oncology, Fudan University Shanghai Cancer Center, Shanghai, China; ^2^ Department of Oncology, Shanghai Medical College, Fudan University, Shanghai, China; ^3^ OrigiMed, Shanghai, China

**Keywords:** metaplastic carcinoma, sarcomatoid carcinoma, carcinosarcomas, KMT2D, TP53, whole-exome sequencing

## Abstract

**Background:** Metaplastic or sarcomatoid carcinomas (MSCs) are rare epithelial malignancies with heterologous histological differentiation that can occur in different organs. The objective of the current study was to identify novel somatically mutated genes in MSCs from different organs.

**Methods:** Whole-exome sequencing was performed in 16 paired MSCs originating from the breast (*n* = 10), esophagus (*n* = 3), lung (*n* = 2), and kidney (*n* = 1). In addition, we collected data on KMT2D mutations from eight independent cohorts (*n* = 195) diagnosed with MSCs derived from the breast (*n* = 83), liver (*n* = 8), esophagus (*n* = 15), lung (*n* = 10), and uterus or ovary (*n* = 79). The expression of KMT2D and its clinical significance were evaluated in our cohort.

**Results:** The most frequently mutated genes were TP53 (13/16, 81%) and KMT2D (5/16,31%). We identified seven somatic KMT2D mutations in the exploratory cohort (*n* = 16 tumors), including three nonsense mutations, two frameshift indels, one missense mutation, and one splice site mutation. Interestingly, two patients showed double hits on KMT2D with nonsense mutations and frameshift indels. In the eight validation cohorts (*n* = 195), the average mutation rates for TP53 and KMT2D were 78% (152/195) and 13% (25/195), respectively. Two or more hits on KMT2D were also present in three validation cohorts. Furthermore, KMT2D mutations were associated with low expression of KMT2D, large tumor size and unfavorable prognosis.

**Conclusions:** These findings provide clues for understanding the genetic basis of MSCs originating from different organs and implicate KMT2D alteration as a frequent pathogenic mutation, allowing provision of appropriate treatment for this rare malignant disease in the future.

## Introduction

Metaplastic breast carcinoma is a rare type of breast cancer and is characterized by the presence of malignant cells showing squamous and/or mesenchymal differentiation (Weigelt et al., 2014; Krings and Chen, 2018). While this disease occurs in the lung (Liu et al., 2016), esophagus (Lu et al., 2018) and kidney (Park et al., 2020), it is frequently designated sarcomatoid carcinoma, which is typically defined by the presence of an epithelial component and a sarcoma-like (mesenchymal) component. For this tumor that occurs in the uterus or ovary, it is called a malignant mixed Müllerian tumor or carcinosarcoma (Jones et al., 2014; Cherniack et al., 2017). Currently, a unified pathologic diagnosis for metaplastic or sarcomatoid carcinomas (MSCs) from different organs is lacking, and the genetic alterations underlying the tumorigenesis of MSCs in different organs are still poorly understood. MSCs typically present at an advanced stage with an aggressive and poorly differentiated nature and do not respond well to chemotherapy or radiotherapy. The overall prognosis is poor, and more effective therapeutic options are needed to treat patients (McCart Reed et al., 2019; Ersek et al., 2020).

Next-generation sequencing shows that TP53 is the most recurrently mutated gene in MSCs (Armstrong et al., 2009; Avigdor et al., 2017; Cherniack et al., 2017; Pécuchet et al., 2017; Lu et al., 2018; Zhang et al., 2019), but TP53 mutations are also frequently detected in other epithelial tumors. Molecular studies on MSCs originating from the breast (Avigdor et al., 2017), uterus or ovary (Gotoh et al., 2019), and liver (Zhang et al., 2019) show that the carcinoma and sarcoma components of MSCs are generally thought to be derived from a single-cell clone, as they have similar genetic alterations. However, it is unclear whether MSCs that exhibit heterologous histological differentiation in different organs have common genetic features.

In this study, we performed whole-exome analyses of 16 cases to determine the genomics of MSCs from different organs. In addition to common mutations in TP53, we found a previously unknown recurrently mutated gene, KMT2D (also known as KMT2B, MLL2 and MLL4), which functions as a major enhancer regulator in various biological processes, including in the regulation of development, differentiation, metabolism, and tumor suppression (Froimchuk et al., 2017). Due to the rarity of these diseases, eight cohorts (*n* = 195) containing KMT2D mutation information were analyzed. The expression of KMT2D and its clinical significance were further evaluated in our cohort. The findings contribute to our understanding of the basic genomics of MSCs that originate from different sites and highlight the histone methyltransferase KMT2D mutation in rare aggressive tumors.

## Materials and Methods

### Patients

Using a database of fresh tissues banked in a period spanning from 2010 to 2018, 16 patient samples diagnosed with MSCs were reviewed by two board-certified pathologists according to the criteria that the neoplasms exhibit heterologous differentiation in histology. Overall survival was defined as the time from surgery to death (June 2020). Signed informed consent was obtained from all patients, and the study was approved by the Clinical Research Ethics Committee of Fudan University Shanghai Cancer Center (Number: 050432-4-1805C).

### Whole–Exome Sequencing

Genomic DNA of each tumor component and matched adjacent normal tissue was extracted separately using a QIAamp DNA Mini Kit (Qiagen) according to the manufacturer’s protocols. DNA from the tumor and matched tissue of the 16 cases was subjected to whole-exome capture using the SureSelect Human All Exon v5 (Agilent) platform and to sequencing on a HiSeq 2000 Genome Analyzer (Illumina). Low-quality reads were excluded by Trimmomatic, the high-quality reads were mapped to the UCSC hg19 reference sequence with BWA (version 0.7.9a). PCR duplicates were removed using Picard (http://picard.sourceforge.net). Somatic variants were detected by Mutect (version 2), and the resulting sequences were analyzed for genomic alterations compared with normal genomic DNA. Annotation of variants was performed using Annovar (version 2017).

### Sanger Sequencing

The primers used for PCR amplification are listed in Supplementary Table 1. PCR amplification was performed using PrimeSTAR Max DNA Polymerase (Takara). PCR products were analyzed by Sanger sequencing. BLAST (http://blast.ncbi.nlm.nih.gov/Blast.cgi) was used for the analysis of sequence data.

### Immunohistochemistry Analysis

Immunohistochemistry (IHC) was performed on 4 μm FFPE tumor slides according to standard procedures. The slides were deparaffinized in xylene and rehydrated. A heat-induced antigen retrieval step was employed using citrate buffer at pH 6.0. Endogenous peroxidase activity was blocked by hydrogen peroxide treatment. Following incubation with protein blocking solution, slides were incubated at 4°C overnight with a KMT2D antibody (1:500, against the KMT2D C-terminus, HPA035977; Sigma-Aldrich). Signal amplification was performed using an IHC detection kit (KIT-5920; Fuzhou Maixin Biotechnology, Fujian, China). Staining was scored for intensity (no staining; weak staining; strong staining).

### Statistical Analyses

Statistical tests were performed using GraphPad Prism 5 (Graphpad Software, La Jolla, CA, United States). Student’s independent sample t-tests were used for comparisons between groups. Fisher’s exact test was used to assess the relationship between KMT2D mutation status and protein expression. The Kaplan-Meier method was used to estimate overall survival curves, and group differences were analyzed by the log-rank test. *p* values were two-tailed, and *p* < 0.05 was considered significant.

## Results

### Whole-Exome Sequencing Was Used to Identify Novel Recurrent KMT2D Mutations in MSCs Originating From Different Organs

**FIGURE 1:**
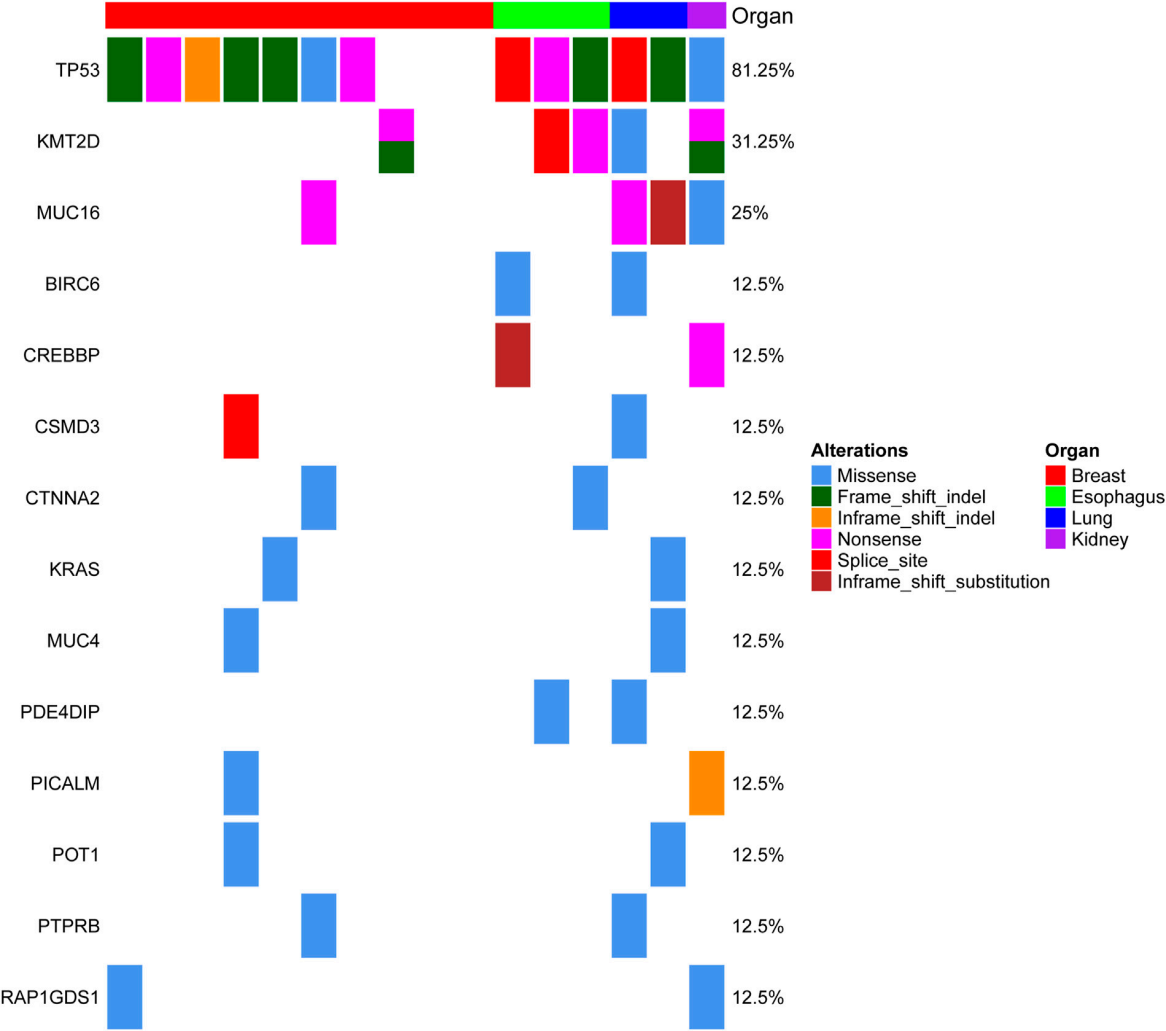
Genomic alterations in MSC from different organs. The matrix represents individual mutations in 16 patient samples originating from four organs (the breast, esophagus, lung and kidney), and the genes with mutations detected in at least two organs are displayed.

**FIGURE 2:**
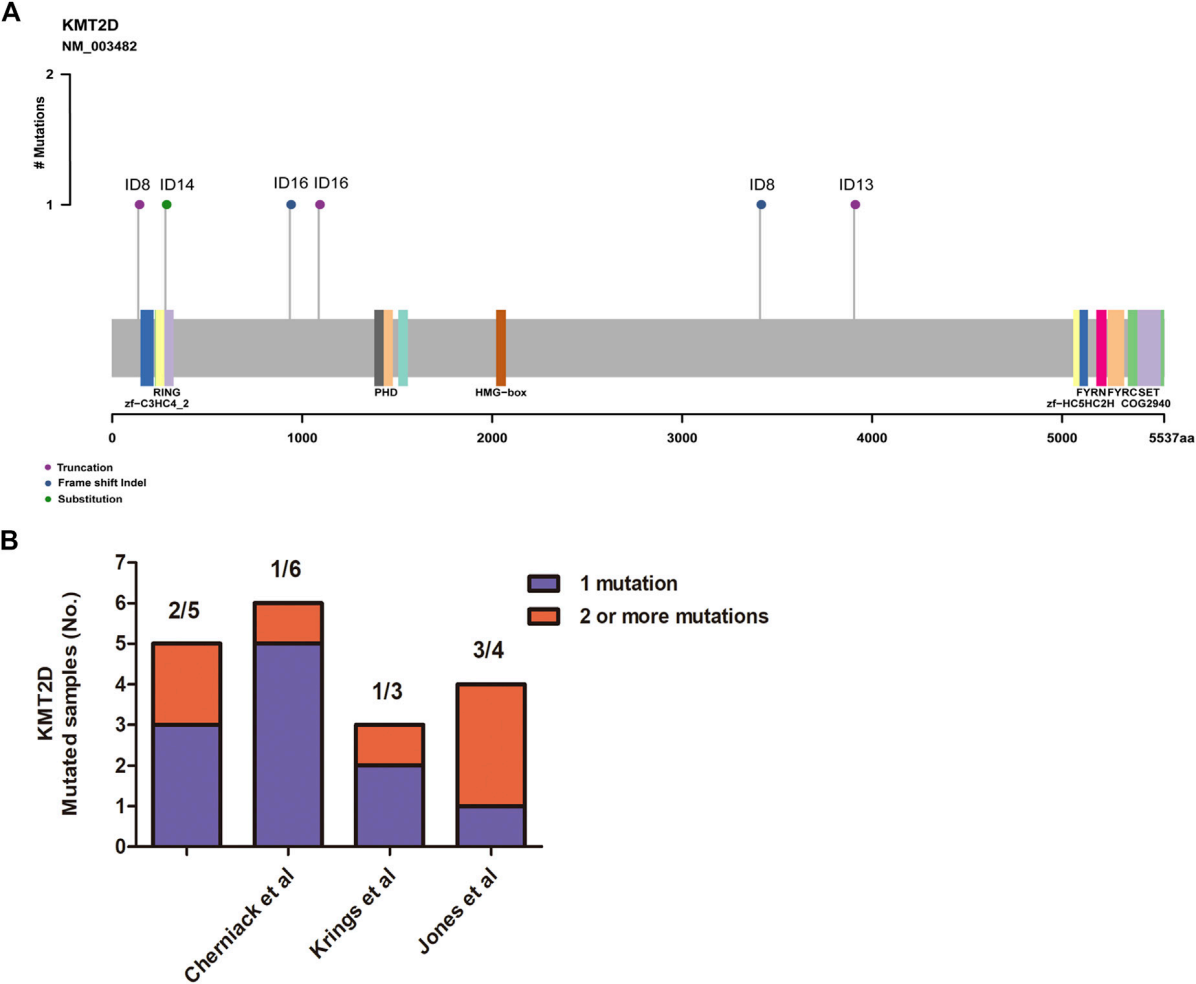
Assessment of the KMT2D mutations. **(A)** Mutation positions in the amino acids of KMT2D. **(B)** The distribution of two or more mutations on KMT2D in our cohort and three other independent cohorts.

MSCs constitute a rare tumor type that has been diagnosed in various organs, including the uterus (Cherniack et al., 2017), breast (Avigdor et al., 2017), lung (Liu et al., 2016), esophagus (Lu et al., 2018), and liver (Zhang et al., 2019). Next-generation sequencing from each study showed that TP53 is the most recurrent mutation gene. Interestingly, MSCs from different organs all exhibited heterologous histological differentiation; therefore, we postulated that specific genetic features might contribute to the tumorigenesis of MSCs. In this study, to define the somatic genetic alterations in MSCs from different sites, 16 paired tumor and matched normal samples (Table 1, 10 pairs from the breast, three pairs from the esophagus, two pairs from the lung, and one pair from the kidney) were subjected to whole-exome sequencing (WES). The mutated genes in 16 MSCs were listed in Supplementary Table 2. Among the 16 tumors, the most frequently mutated genes were TP53 (13/16), KMT2D (5/16), MUC16 (4/16), and PIK3CA (4/16) (Supplementary Figure 1). Consistent with the current studies (Avigdor et al., 2017; Cherniack et al., 2017; Lu et al., 2018; Zhang et al., 2019) in the genomics of MSCs, TP53 mutation was the most common genetic alteration. In addition to TP53, other recurrently mutated genes in breast MSCs were PIK3CA (4/10) and PTEN (2/10); however, in other MSCs, we observed mutations in KMT2D (4/6), MUC16 (3/6), BCLAF1 (2/6), BIRC6 (2/6), and CREBBP (2/6). Additionally, we further searched for common mutations in MSCS from different organs, which may be driver mutations in the tumorigenesis of MSCs. As shown in Figure 1, gene mutations occurring in at least two organs (breast, esophageal, lung, and kidney) were found, and the results also showed that TP53 and KMT2D were the most frequently mutated genes. For KMT2D, which is rarely reported to be associated with MSCs, we further confirmed that the KMT2D gene was specifically mutated in tumors, not in paired normal tissues, by PCR and Sanger sequencing (Supplementary Figure 2). We identified seven somatic KMT2D mutations in the exploratory cohort (*n* = 16), including three nonsense mutations, two frameshift indels, one missense mutation, and one splice site mutation (Figure 2A, Table 1).

**TABLE 1:** Characteristics of patients.

Case ID	Gender	Age	Organ	Differentiation	Size	KMT2D mutation
1	Female	46	Breast	Spindle	3.5	No
2	Female	48	Breast	Spindle	3.7	No
3	Female	46	Breast	Spindle	3.5	No
4	Female	63	Breast	Spindle	2.0	No
5	Female	67	Breast	Spindle	2.0	No
6	Female	66	Breast	Osseous	6.0	No
7	Female	64	Breast	Spindle	2.2	No
8	Female	44	Breast	Osseous	12.0	c.10229delC (f.d), c.G410A (stop-gain)
9	Female	42	Breast	Spindle	3.3	No
10	Female	43	Breast	Squamous	3.0	No
11	Male	66	Esophagus	Spindle	7.0	No
12	Male	66	Esophagus	Spindle	8.0	c.5533+1G>A (splicing)
13	Male	58	Esophagus	Spindle	4.0	c.C11713T (stop-gain)
14	Male	49	Lung	Spindle	6.0	c.G839T (nonsynonymous)
15	Male	71	Lung	Spindle	6.0	No
16	Male	69	Kidney	Spindle	13.0	c.G3259T (stop-gain), c.2803delC (f.d)

Size: tumor diameter (cm); f.d, frameshift deletion.

### KMT2D Mutations in the Eight Validation Cohorts

MSCs exhibited frequent KMT2D mutations in the exploratory cohort (*n* = 16 tumors). Due to the rarity of these diseases, we sought to determine whether KMT2D mutations existed in expanded cohorts. We searched the literature and cBioPortal database with information on KMT2D mutation in MSCs from different organs. As listed in Table 2, six studies and two uterine carcinosarcoma datasets (http://cbioportal.org) containing KMT2D mutation information were available for analysis. The eight cohorts (*n* = 195) included three cohorts derived from the breast (*n* = 35 (Ng et al., 2017), *n* = 28 [2], *n* = 20 (Ross et al., 2015)), one cohort (Zhang et al., 2019) derived from the liver (*n* = 8), one cohort (Lu et al., 2018) derived from the esophagus (*n* = 15), one cohort (Liu et al., 2016) derived from the lung (*n* = 10), and two cohorts derived from the uterus or ovary [*n* = 57 (Cherniack et al., 2017), *n* = 22 (Jones et al., 2014)]. Consistent with our studies, the eight validation cohorts (Table 2) showed that MSCs harbored high TP53 mutations (from 60 to 100%, average, 78%). For KMT2D (Table 2), the mutation rate ranged from 6 to 30% (average, 13%), and the mutation type contained truncation, frameshift, splice and missense mutations. Interestingly, some patients simultaneously showed two or more mutations in KMT2D. In our study, two patients (ID8 and ID 16) showed two mutations in KMT2D with nonsense mutations and frameshift indels (Figure 2A). Consistent with our study, two or more hits on KMT2D were also present in the other three independent cohorts (Figure 2B).

**TABLE 2:** The mutations of TP53 and KMT2D in the eight cohorts.

Organ	Sequencing	Total cases	TP53 Cases	Mutations for KMT2D					Literature
				Cases	Truncation (No.)	Frame-shift (No.)	Splice (No.)	Missense (No.)	
Breast	Whole-exome	35	24 (69%)	2 (6%)	1	1	0	0	Ng et al.
Breast	panel	28	18 (64%)	3 (11%)	2	2	0	0	Krings et al.
Breast	panel	20	15 (75%)	6 (30%)	6	0	0	0	Ross et al.
Liver	panel	8	6 (75%)	1 (13%)	0	0	1	0	Zhang et al.
Esophagus	panel	15	15 (100%)	2 (13%)	0	0	0	2	Lu et al.
Lung	Whole-exome	10	6 (60%)	1 (10%)	0	0	0	1	Liu et al.
Uterus	Whole-exome	57	52 (91%)	6 (11%)	0	2	0	6	Cherniack et al.
Uterus and ovary	Whole-exome	22	16 (73%)	4 (18%)	2	2	1	6	Jones et al.

### KMT2D Mutations Are Negatively Associated With KMT2D Expression

**FIGURE 3:**
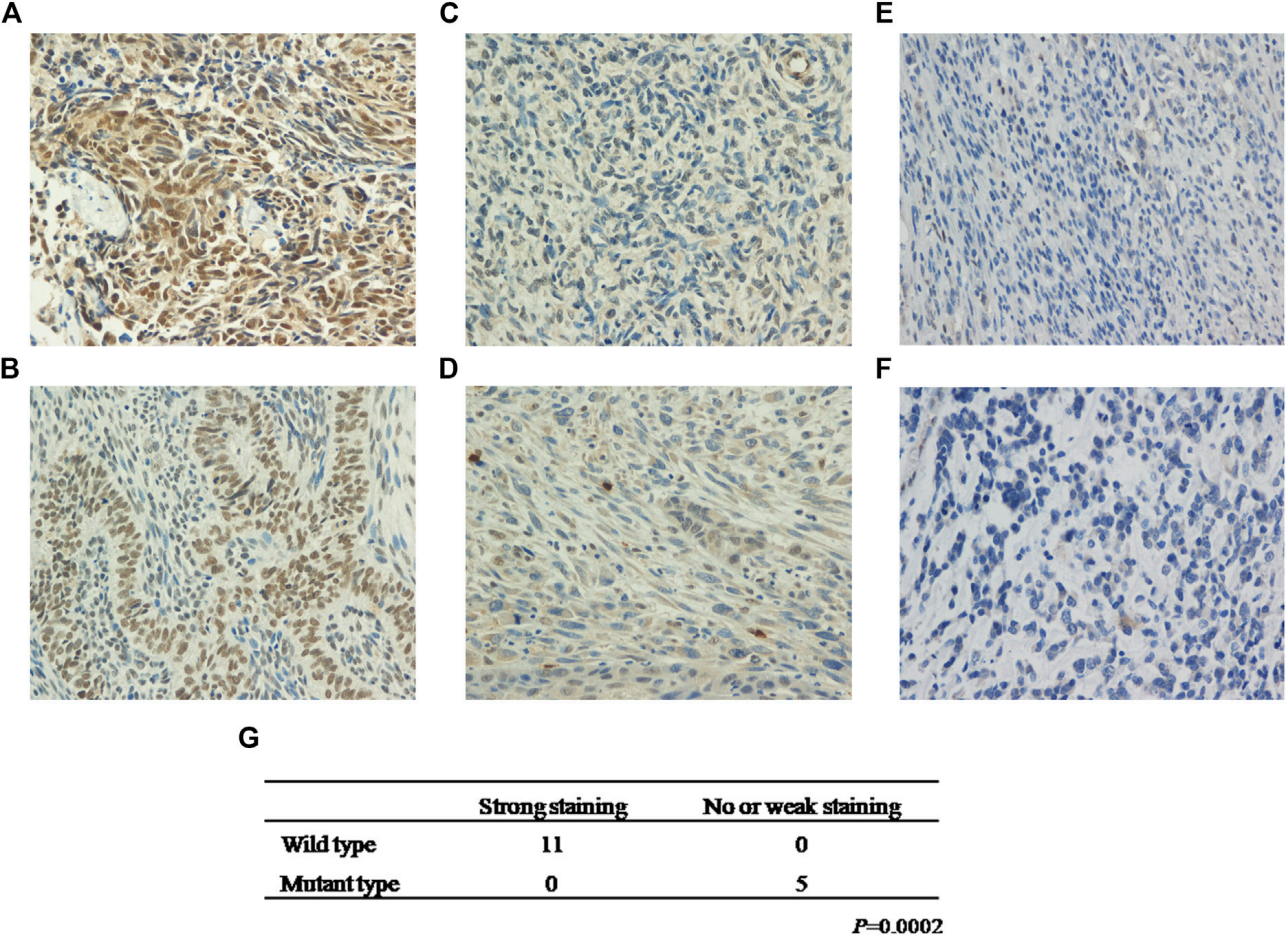
KMT2D mutations are negatively associated with KMT2D expression. Strong staining of KMT2D in MSCs with no KMT2D mutation originating from the breast (**A**, Patient ID 2) and esophagus (**B**, Patient ID 11). Week staining of MSCs with a single mutation in KMT2D originating from the lung (**C**, Patient ID 14) and esophagus (**D**, Patient ID 13). Loss of KMT2D expression in MSCs with double mutations in KMT2D originating from the breast (**E**, Patient ID 8) and kidney (**F**, Patient ID 16). **(G)** The relationship between KMT2D mutation status and protein expression.

To establish the relationship between KMT2D mutation status and protein expression, we examined KMT2D expression in the exploratory cohort (*n* = 16 tumors) by IHC. Interestingly, KMT2D showed robust nuclear expression in patients with no KMT2D mutation (*n* = 11) (Figures 3A,B), whereas a weak KMT2D signal was detected in patients with a single mutation in KMT2D (*n* = 3) (Figures 3C,D); moreover, the two cases with double mutations in KMT2D exhibited loss of nuclear KMT2D expression (Figures 3E,F). Our results showed that KMT2D mutations were significantly associated with low expression of KMT2D (Figure 3G).

### Association Between KMT2D Mutation and Prognosis

**FIGURE 4:**
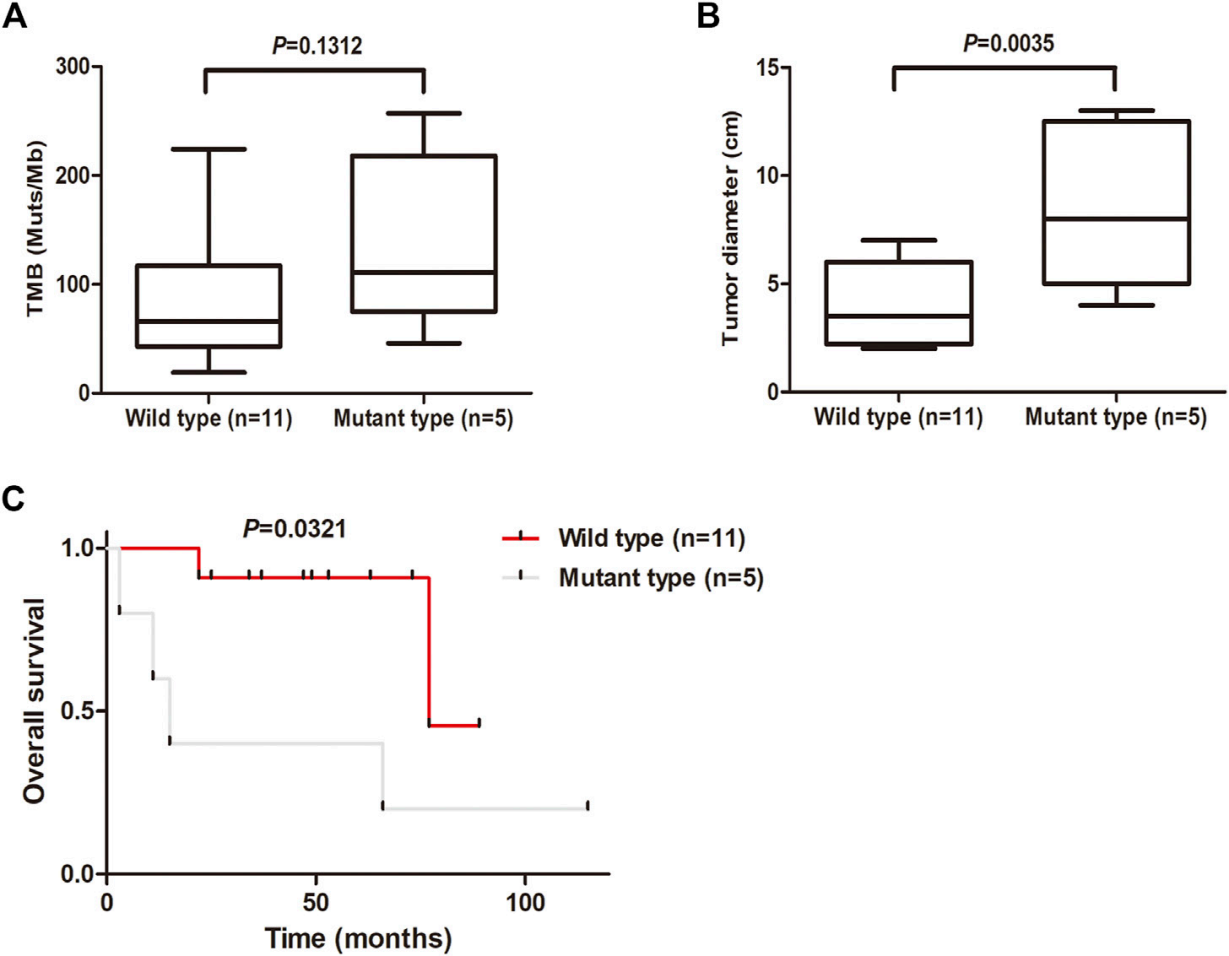
KMT2D mutations are associated with large tumor size and unfavorable prognosis. **(A)** Comparison of TMB (mutations/MB) between the KMT2D wild-type group and mutant-type group. **(B)** Comparison of tumor diameter between the KMT2D wild-type group and mutant-type group. **(C)** Survival analysis for patients between the KMT2D wild-type group and mutant-type group.

The tumor mutation burden (TMB) was defined as the number of nonsynonymous and indel mutations per megabase (Mb). The patients with MSC showed high TMB, ranging from 19 to 257, with a median of 80 (Figure 4A). Notably, there was a tendency for patients with KMT2D mutations to show high TMB, although in this study the difference was not significant (Figure 4A). We further analyzed the relationships between KMT2D and tumor size and prognosis. The results showed that the tumor size in MSC patients with KMT2D mutations was significantly higher than that in patients without KMT2D mutations (*p* = 0.0035) (Figure 4B). Furthermore, KMT2D mutations were associated with shorter overall survival times (*p* = 0.0321) (Figure 4C).

## Discussion

MSCs are rare neoplasms with heterologous histological differentiation that can occur in different organs (Weigelt et al., 2014; Liu et al., 2016; Cherniack et al., 2017; Krings and Chen, 2018; Lu et al., 2018; Park et al., 2020). To identify novel somatically mutated genes in MSCs from different organs, we performed WES on 16 primary MSCs and paired normal DNA. The results showed that in addition to TP53 (13/16), KMT2D (5/16) was a frequently mutated gene. Another eight cohorts (*n* = 195) diagnosed with MSCs originating from six organs also showed high mutations in KMT2D (6–30%, with an average of 13%). Our finding that KMT2D is somatically mutated in 31% of MSCs and is partially displayed by frameshift, nonsense mutations, and dual mutations strongly implicates KMT2D as a novel pathogenic driver gene in MSCs.

Exome sequencing of 10 cases with breast MSCs confirmed enrichment of TP53 (7/10) and PIK3CA (4/10) (Supplementary Figure 1). Consistent with our results, breast MSCs harbored high TP53 mutations in three independent cohorts (69, 64, and 75%; Table 2). Interestingly, TP53 was more frequently mutated in breast MSCs than in breast cancers. Pereira et al. (Pereira et al., 2016) sequenced 173 genes in 2,433 primary breast tumors, and PIK3CA and TP53 dominated the mutation landscape (40.1 and 35.4%, respectively). Similar results were also described in another cohort (*n* = 1918, http://cbioportal.org) (Razavi et al., 2018). These results raise the possibility that the mutation of TP53 may be strongly associated with the tumorigenesis of MSCs. In addition to breast MSCs, we found that TP53 and KMT2D were the most frequently mutated genes (6/6 and 4/6, respectively) in MSCs originating from the other three organs (esophagus, lung and kidney) (Figure 1); specifically, each tumor (*n* = 6) exhibited a TP53 mutation. Moreover, we also found that TP53 in the liver, esophageal, lung, uterine and ovarian MSCs was prone to mutation, and the mutation rate ranged from 60 to 100% (average 84.8%) (Table 2). The KMT2D mutation, which is rarely reported to be involved in MSCs, was frequently detected in our study (5/16) (Table 1). To our knowledge, our study is the first to report that KMT2D is a recurrently mutated gene in MSCs from different organs. Notably, small cell lung cancer exhibits high mutation rates of TP53 and KMT2D. Ross et al. showed that the genomic alteration rates in TP53 and KMT2D were 86 and 17%, respectively, in 98 cases (Ross et al., 2014); in the Hu study, the mutation frequencies for TP53 and KMT2D were 93.4 and 15.6%, respectively, in 122 patients with small cell lung cancer (Hu et al., 2019). TP53 mutants engage in direct cross talk with chromatin regulatory genes (KMT2A, KMT2D and KAT6A), resulting in genome-wide transcription programs (Zhu et al., 2015). Together with our findings, these results indicate that both MSCs and small cell lung cancer have similar mutation rates for TP53 and KMT2D, and the mutation of KMT2D may cooperate with TP53 mutation to drive tumorigenesis in MSCs and small cell lung cancer.

Studies in mouse models highlight the importance of KMT2D in regulating a wide range of biological processes, including embryonic development, cell differentiation, metabolic homeostasis, and cancer (Froimchuk et al., 2017). Pathogenic variants in KMT2D cause autosomal dominant Kabuki syndrome, a disorder characterized by distinctive facial features, intellectual disability, and abnormalities affecting other parts of the body (Cocciadiferro et al., 2018). KMT2D mutations result in an abnormal and nonfunctional lysine-specific methyltransferase 2D enzyme that disrupts its role in histone methylation and impairs proper activation of certain genes, resulting in abnormalities in many of the body’s organs and tissues (Froimchuk et al., 2017). Mutations in the KMT2D gene have been implicated in certain cancers, including medulloblastomas (Dhar et al., 2018), lymphomas (Zhang et al., 2015) and lung cancer (Alam et al., 2020). With this study, we are the first to report that KMT2D is one of the most recurrently mutated genes (5/16) in MSCs originating from different organs (Figure 1). The KMT2D mutations were represented by three nonsense mutations, two frameshift indels, one missense mutation, and one splice site mutation in the exploratory cohort (*n* = 16 tumors) (Table 1). Interestingly, two patients (ID 8 and ID 16) each showed two mutations in KMT2D, a nonsense mutation and frameshift indel (Figure 2A). Consistent with our results, the eight validation cohorts showed that MSCs harbored frequent KMT2D mutations ranging from 6 to 30% (average of 13%) (Table 2). Two or more mutations of KMT2D in one patient were simultaneously detected in three independent cohorts (Figure 2B). Moreover, we found that KMT2D mutations were negatively associated with KMT2D expression (Figure 3). Frequent KMT2D mutations in MSCs point to their loss of function in pathogenesis and suggest their roles as tumor suppressors. KMT2D loss led to increased DNA damage and mutation burden, chromatin remodeling, intron retention, activation of transposable elements and overall instability at the chromosomal level (Kantidakis et al., 2016). This type of stress can have catastrophic consequences and lead to the formation of cancerous tumors. Consistent with these outcomes, lung-specific loss of KMT2D promotes lung tumorigenesis in mice and upregulates the glycolysis program (Alam et al., 2020). Interestingly, KMT2D-mutant tumors exhibit enhanced immune infiltration in the tumor microenvironment, and KMT2D deficiency sensitizes multiple cancer types to anti-PD1 therapy by augmenting tumor immunogenicity (Wang et al., 2020). In summary, KMT2D is frequently mutated in MSCs from different organs, and KMT2D alteration may act as a driver mutation and provide a basis for anti-PD1 therapy in this rare malignant disease.

To our knowledge, this is the first study to specifically investigate genomic alterations in MSCs derived from different organs. We found that TP53 and KMT2D were the most recurrently mutated genes. In addition, KMT2D alterations presented with nonsense mutations, frameshift indels, missense mutations, and splice site mutations, and some patients harbored double hits on KMT2D. Similar results were also seen in eight validation cohort studies. Furthermore, KMT2D mutations were associated with low expression of KMT2D, large tumor size and unfavorable prognosis. Collectively, these findings lead us to identify KMT2D as a pathogenic driver gene in MSCs derived from different organs, which may lead to appropriate treatments for this rare malignant disease in the future.

## Data Availability

The summaries of the data presented in this study are shown in Supplementary Table 2, and the data generated for this study are available on request to the corresponding authors.
