# Supplementary material for: Genomic Analyses of Metaplastic or Sarcomatoid Carcinomas From Different Organs Revealed Frequent Mutations in KMT2D
Source: Front Mol Biosci. 2021 Jul 15;8:688692. doi: 10.3389/fmolb.2021.688692 (PMC8319738; doi:10.3389/fmolb.2021.688692)
Supplement: Supplementary file 2 [file Table1.docx]

| **Number** | **Mutation location** | **Primer** |
| --- | --- | --- |
| 1 | chr12: 49430910 | F: 5' TTTCTGCCTCTACCACTAGTATGAT 3' |
| 1 | chr12: 49430910 | R: 5' ACAGCCAGTGCTATCACAGAAG 3' |
| 2 | chr12: 49448190 | F: 5' CCCAGTATACCCATGGTCCTT 3' |
| 2 | chr12: 49448190 | R: 5' ACCTGCCCACCTAGGAGAAC 3' |
| 3 | chr12: 49437145 | F: 5' CCAGCCTCCAACACTGACAA 3' |
| 3 | chr12: 49437145 | R: 5' TGTGGGACTGGAGACCTGTTC 3' |
| 4 | chr12: 49426775 | F: 5' GCTGAGGAGACAGTAAAGTTCGA 3' |
| 4 | chr12: 49426775 | R: 5' CTGATGTCACACAGTGGGCA 3' |
| 5 | chr12: 49447259 | F: 5' AGGTGCACATGGAACATTACAGT 3' |
| 5 | chr12: 49447259 | R: 5' CTGTACCAGCTGTGGGCATC 3' |
| 6 | chr12:49444112 | F: 5' CCTAGGCCAGAGAAGTCATCC 3' |
| 6 | chr12:49444112 | R: 5' CTACCACTGTCCGTTCCCTC 3' |
| 7 | chr12:49444568 | F: 5' AGTGCAGTTAGCTTCTGGTGGA 3' |
| 7 | chr12:49444568 | R: 5' GGTAAGGGGATGTTAGTACTCTGTT 3' |

**Supplementary Table 1: Primers for KMT2D mutations**
