# Supplementary material for: Genomic Analyses of Metaplastic or Sarcomatoid Carcinomas From Different Organs Revealed Frequent Mutations in KMT2D
Source: Front Mol Biosci. 2021 Jul 15;8:688692. doi: 10.3389/fmolb.2021.688692 (PMC8319738; doi:10.3389/fmolb.2021.688692)

Supplementary Figure 2. Sanger sequencing validation of the KMT2D mutations. KMT2D mutations were specifically detected in tumors. A: Patient ID 13 with stop-gain mutation. B: Patient ID 14 with nonsynonymous mutation. Patient ID 8 with frameshift deletion (C) and stop-gain mutation (D).

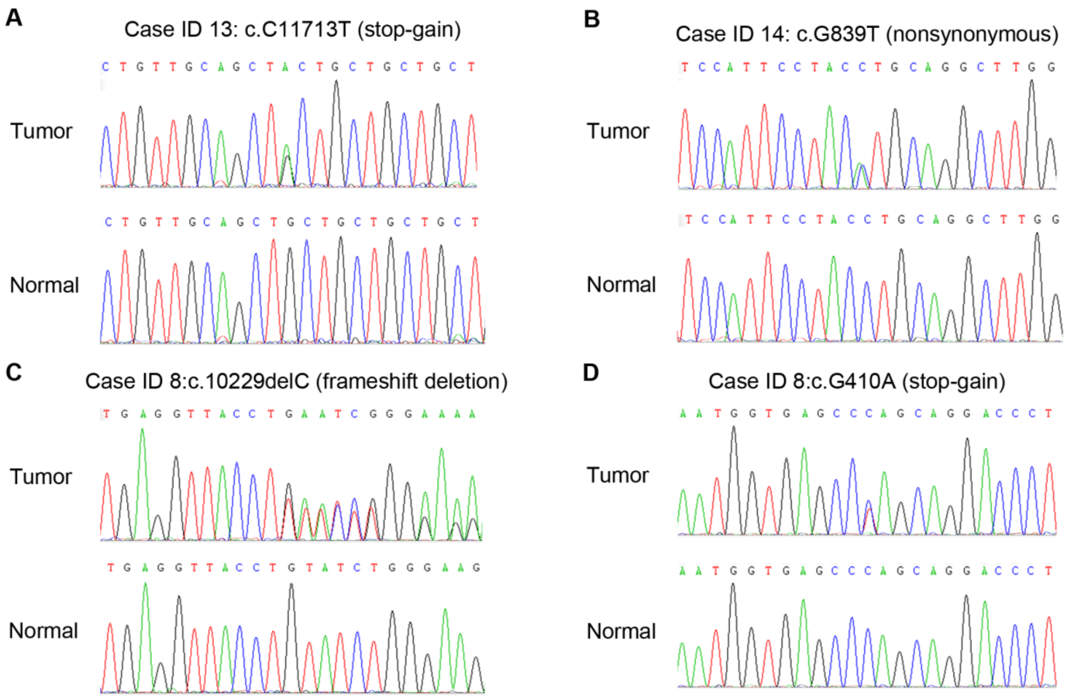

Supplement: Supplementary file 3 [file Image2.pdf]
