# Supplementary material for: Genomic Analyses of Metaplastic or Sarcomatoid Carcinomas From Different Organs Revealed Frequent Mutations in KMT2D
Source: Front Mol Biosci. 2021 Jul 15;8:688692. doi: 10.3389/fmolb.2021.688692 (PMC8319738; doi:10.3389/fmolb.2021.688692)

Supplementary Figure 1. Genomic alterations in MSCs from different organs. The matrix represents individual mutations in 16 patient samples originating from four organs (breast, esophagus, lung and kidney).

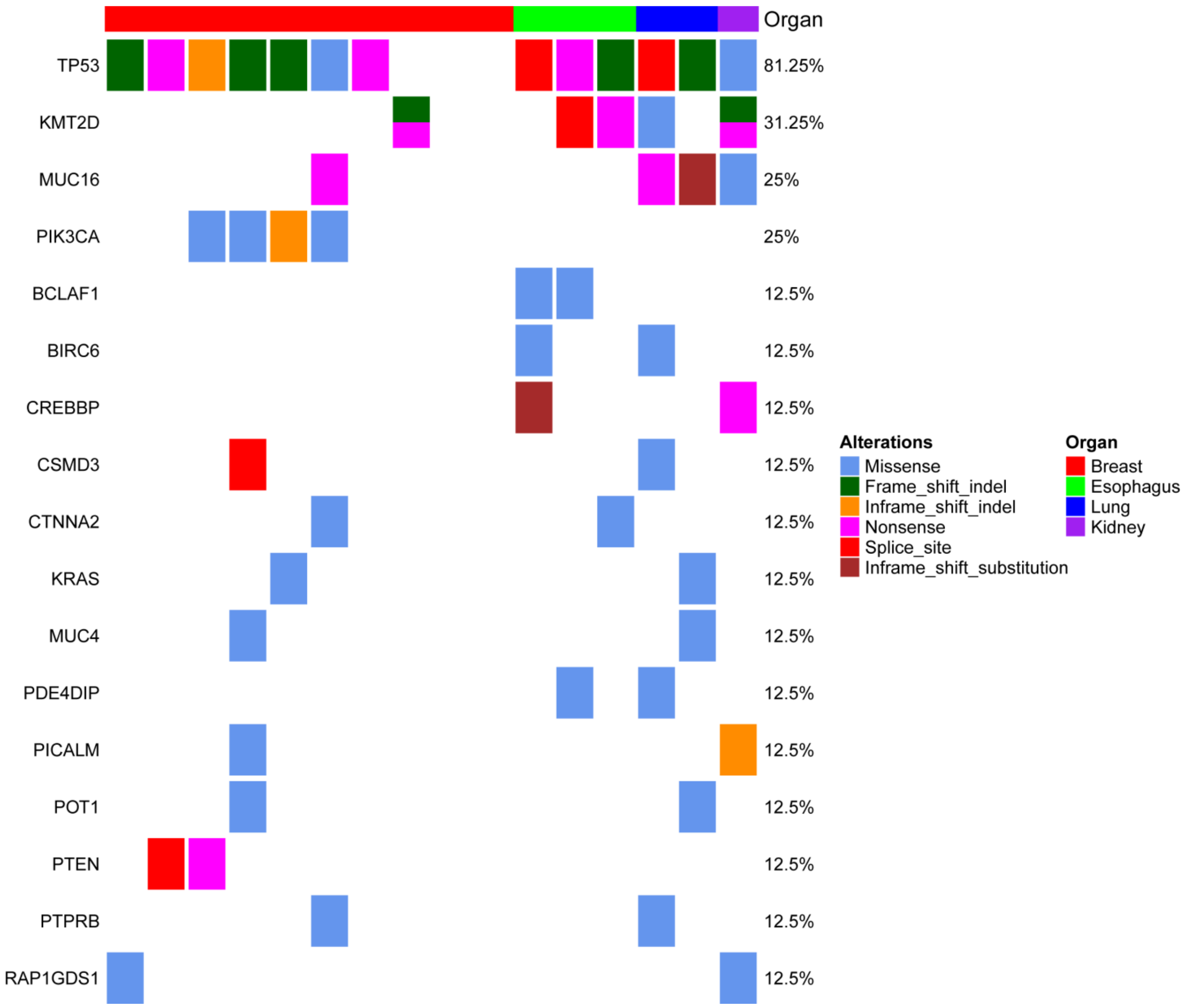

Supplement: Supplementary file 4 [file Image1.pdf]
